# Methicillin-Resistant *Staphylococcus aureus*, Samoa, 2007–2008

**DOI:** 10.3201/eid1706.101083

**Published:** 2011-06

**Authors:** James Alesana-Slater, Stephen R. Ritchie, Helen Heffernan, Tracy Camp, Alice Richardson, Peter Herbison, Pauline Norris

**Affiliations:** Author affiliations: University of Otago, Dunedin, New Zealand (J. Alesana-Slater, P. Herbison, P. Norris);; University of Auckland, Auckland, New Zealand (S.R. Ritchie);; Institute of Environmental Science and Research Limited, Wellington, New Zealand (H. Heffernan, A. Richardson);; Auckland City Hospital, Auckland (T. Camp)

**Keywords:** Staphylococcus aureus, methicillin-resistant Staphylococcus aureus, wound infection, soft tissue infection, drug resistance, bacteria, Samoa, research

## Abstract

Little is known about the epidemiology of methicillin-resistant *Staphylococcus aureus* (MRSA) in most Pacific Island nations. Relatively high rates of MRSA have been reported in Polynesian people living outside the Pacific Islands. To determine the prevalence and characteristics of MRSA, we assessed wound swabs from 399 persons with skin and soft tissue infection living in Samoa. MRSA was isolated from 9% of study participants; 34 of the 196 *S. aureus* isolates were MRSA. Five MRSA genotypes were identified; the 3 most common were USA300, the Queensland clone, and a sequence type 1 MRSA strain that shares <85% homology with the sequence type 1 MRSA strain common in the region (WA MRSA-1). The Southwest Pacific MRSA clone was identified but accounted for only 12% of MRSA isolates. The high prevalence of MRSA in Samoa provides impetus for initiatives to improve antimicrobial drug resistance surveillance, infection control, and antimicrobial drug use in Pacific Island nations.

## Abstract 2

TOC Summary: A wide range of MRSA genotypes cause wound infections.

Infections caused by methicillin-resistant *Staphylococcus aureus* (MRSA) have become a global health concern during the past 2–3 decades. The epidemiology of MRSA has demonstrated marked geographic variation in the prevalence and genotypes of MRSA (*1*,*2*), and recent reports from many parts of the world indicate that the prevalence and diversity of MRSA continue to increase (*3*,*4*). Studies of the global epidemiology frequently have not included MRSA obtained from persons living in developing nations. The prevalence and genetic variation of MRSA infection in most Pacific Island nations remain unknown. The only study of MRSA in the Pacific was conducted after a report in 2004 of an increased incidence of MRSA infection in Polynesian people in Hawaii (*5*). This study showed that most MRSA infections in Hawaii were caused by the USA300 MRSA strain (*6*).

Relatively high rates of MRSA infection have been reported in Polynesian people living outside the Pacific Islands region, including in Alaska, Australia, and New Zealand, and have been attributed to infection with the Southwest Pacific clone of MRSA (sequence type [ST] 30 SCC*mec* type IV, also referred to as Western Samoan phage pattern [WSPP] MRSA or the Oceania strain) (*7*–*10*). The Southwest Pacific clone of community-associated MRSA was identified in New Zealand in 1992 (*11*). The first isolate and many subsequent isolates of this strain were from persons in New Zealand who had some association with Samoa. The Southwest Pacific MRSA clone has now spread as far as Europe and South America (*4*,*12*). This MRSA strain has been postulated to have arisen from a pandemic penicillin-resistant *S. aureus* strain, known as phage type 80/81, that caused serious hospital- and community-acquired infections during the 1950s (*13*).

Samoa is an independent nation in the Southwest Pacific, with a population of ≈180,000 persons (Samoan Statistics Department, www.spc.int/prism/wstest/index.htm; Figure). Samoa has a small and developing economy predominantly comprising remittances from Samoan persons living overseas, agriculture, and tourism. The gross domestic product per capita was US $2,987.90 in 2008 (http://data.un.org). Life expectancy in Samoa is 74.9 years for women and 68.5 years for men, and the infant mortality rate is ≈22.3 per 1,000 live-born infants. The publicly funded National Health Service is based at the national referral hospital in Apia; in addition, Samoa has 7 district hospitals (*14*). Several outreach and integrated community health services provide primary health care services, such as clinics and vaccinations.

**Figure:**
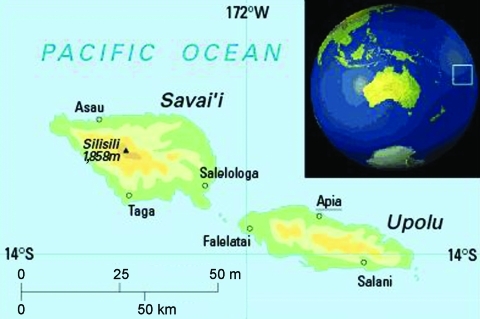
Map of Samoa, showing the 2 main islands, Upolu and Savai’i, and the capital Apia. Reproduced with permission from Oxford Cartographers (www.oxfordcartographers.com).

Samoa gained independence from New Zealand in 1962; a large Samoan population resides in New Zealand. In New Zealand, infections caused by *S. aureus*, whether methicillin susceptible or resistant, are more common in Maori and Polynesian persons than in other ethnic groups (*15*–*17*). Antimicrobial drug resistance is routinely monitored in New Zealand through testing of bacterial isolates from specimens taken for diagnostic purposes. In 2008, 9% of *S. aureus* isolates were methicillin resistant, but this rate varied considerably among different parts of the country, with rates as high as 13%–16% in hospitals in the Auckland area (*18*).

In Samoa, as in many other developing countries, antimicrobial drug resistance is not systematically monitored. The microbiology service at the main public hospital in Apia performs antimicrobial drug susceptibility testing only when specifically requested by the treating doctor. This testing is infrequent, and the results are not regularly collated (V. Kini, pers. comm.). Testing for antimicrobial drug susceptibility requires equipment, resources, and a high level of expertise and quality assurance—requirements that are beyond the means of many laboratories in the developing world. If a high rate of antimicrobial drug resistance exists in Samoa, it would substantially affect this relatively poor country with relatively high rates of infectious diseases. We performed a systematic study to assess the prevalence and characteristics of MRSA isolated from wound swabs from persons with skin and soft tissue infection living in Samoa.

## Methods

During the summer of 2007–2008, a study investigator (J.A.S.) collected *S. aureus* isolates from persons with skin and soft tissue infection. Review of patient notes was used to identify hospital patients who had skin lesions. All ambulatory patients who attended a clinic during the visit by J.A.S. and any family members who accompanied them were asked if they had a skin lesion. All persons with a skin lesion were invited to participate in the study; <5 persons declined to participate. At hospitals, participants included inpatients and outpatients, but this information was not recorded. No attempt was made to categorize infections as hospital acquired or community acquired, although the source of the wound was recorded.

All participants had skin and soft tissue infection, but this infection was not always the primary reason for contact with health services. Skin and soft tissue infection was defined as increasing pain, warmth, induration, erythema, or purulent discharge. Swabs were collected twice from patients who had separate lesions on the upper and lower body. If a patient had >1 infected site in the same region (e.g., upper body), only the larger lesion was swabbed.

Patient data collected included demographic information (age, sex, occupation, and village of residence) and recent exposure to health services (use of antimicrobial drugs in the last month and contact with health services in last 3 months). We attempted to include persons of different ages, both sexes, and various occupations.

The Lower South Ethics Committee in New Zealand, and the Health Research Council in Samoa gave ethical approval for the study. Participants were given an information sheet about the research and a small card explaining, in Samoan, how to take care of wounds. The study investigator explained the purpose of the study in Samoan, and the consent form was printed in Samoan and in English.

Swabs were taken after the wound was cleaned with sterile water. Swabs were placed into Amies transport media (Fort Richard Laboratories, Auckland, New Zealand), stored on ice, and transported to the laboratory at 1 of the 2 main hospitals. All swabs were processed within 12 hours after collection. Swabs were spread onto sheep blood agar containing aztreonam (6 mg/L) and incubated for 16–24 hours. A catalase test was performed on colonies suspected to be *S. aureus*, and catalase-positive colonies were identified by using a latex agglutination test (BBL Staphyloslide Latex Test, Becton Dickinson, Sparks, MD, USA). *S. aureus* isolates were transported on a nutrient agar slope to LabPLUS (Auckland, New Zealand).

In New Zealand, antimicrobial drug susceptibility testing was performed by using disk diffusion for penicillin, and agar dilution breakpoint testing for cotrimoxazole, doxycycline, erythromycin, gentamicin, oxacillin, and vancomycin in accordance with Clinical and Laboratory Standards Institute methods and interpretive standards (*19*–*21*). MRSA isolates were tested by disk diffusion for susceptibility to ciprofloxacin and clindamycin, including inducible clindamycin resistance. Additional fusidic acid and mupirocin disk diffusion susceptibility testing was performed when required to assist with identification of MRSA strains.

MRSA isolates were tested for the genes encoding for Panton-Valentine leukocidin (PVL) by PCR (*22*). Multilocus sequence typing (*23*); *spa* typing (*24*); and if necessary, pulsed-field gel electrophoresis (PFGE) of *Sma*I-digested genomic DNA were used to identify and characterize MRSA strains. StaphType software version 1.5 (Ridom GmbH, Würzburg, Germany) was used to assign *spa* types. We compared *spa* types by using the BURP (based upon repeat pattern) algorithm and excluding *spa* types with <5 repeats and setting a maximum cost of 4 between members of a *spa* group cluster. PFGE banding patterns were analyzed with BioNumerics software version 5.1 (Applied Maths, Saint-Martens-Latern, Belgium) by using the Dice coefficient and unweighted pair group method with arithmetic averages, at settings of 0.5% optimization and 1.5% position tolerance.

Categorical data are expressed as proportions and 95% confidence intervals (CIs), calculated by the modified Wald method. Statistical significance of comparative categorical data was examined by using the χ^2^ test.

## Results

A total of 423 swabs were obtained from skin and soft tissue infections of 399 persons at 8 locations on the 2 main islands of Samoa (Upolu and Savai’i) and a smaller island, Manono. The 8 locations were the main public hospital in Apia (the capital on Upolu), the main public hospital on Savai’i, 5 district hospitals around the main islands, and community-based clinics on Manono.

Infections in skin and soft tissue were in wounds from falls, cuts, dog bites, car accidents, and burns; as well as boils and carbuncles. Many were described as *po’u*, a Samoan term for idiopathic tropical ulcer. Twenty-four persons each had swabs from 2 separate lesions. Even though we did not categorize infections as hospital acquired or community acquired, we were able to infer likely place of acquisition from the description of the wound: 20 (5%) of 399 represented infections of surgical sites, which were hospital acquired; 335 (84%) represented community-acquired infections, such as impetigo; *po’u*; boils; or infections of wounds resulting from dog bites, machete cuts, or assaults. We were not able to further classify the remaining 44 (11%) infections.

*S. aureus* was isolated from 196 (46%, 95% CI 42%–51%) of the 423 wound swabs and from 187 (47%, 95% CI 42%–52%) of the 399 study participants. *S. aureus* was more likely to be obtained from children <5 years of age than from persons in other age groups (37 [70%] of 53 compared with 150 [43%] of 346; p<0.001) and less likely in persons currently or recently treated with antimicrobial drugs (84 [38%] of 224, compared with 103 [59%] of 175 persons who were not; p<0.001) (Table 1).

**Table 1:** Demographic characteristics of study participants and prevalence of MSSA and MRSA, Samoa, summer 2007–2008*

Characteristic	Total study population	No. (%, 95% CI)		
		*S. aureus* positive	MSSA	MRSA
No. participants	399	187 (47, 42–52)	153 (38, 34–43)	34 (9, 6–12)
Male sex	263	121 (46, 40–52)	95 (36, 31–42)	26 (10, 7–14)
Age, y†				
<5	53	37 (70, 56–81)‡	33 (62, 49–74)	4 (8, 3–18)
5–15	93	50 (54, 44–64)	44 (47, 38–57)	6 (7, 3–14)
16–59	195	79 (34–48)	60 (31, 25–38)	19 (10, 6–15)
>60	58	21 (36, 25–49)	16 (28, 18–40)	5 (9, 3–19)
Antimicrobial drug treatment in previous month	224	84 (38, 31–44)§	67 (30, 24–36)	17 (8, 5–12)
Health care contact in previous 3 months	262	108 (41, 35–47)	85 (32, 27–38)	23 (9, 6–13)

*MSSA, methicillin-susceptible *Staphylococcus aureus*, MRSA, methicillin-resistant *S. aureus*; CI, confidence interval. †Median age, y (range) of study participants: All, 32 (0–89); *S. aureus* positive, 24 (0–84); MSSA positive, 26 (0–84); MRSA positive, 36 (0–76). ‡p<0.001, *S. aureus* positive vs. study population >5 y of age. §p<0.001, *S. aureus* positive vs. no antimicrobial drug treatment in the previous month.

Thirty-four (17%, 95% CI 13%–23%) of the 196 *S. aureus* isolates were methicillin resistant. MRSA was isolated from 8% (95% CI 6%–11%) of the 423 wound swabs and from 9% (95% CI 6%–12%) of the 399 study participants. Almost 1 in 5 of the 187 participants with *S. aureus* had MRSA (34/187, 18%, 95% CI 13%–24%). The basic demographic features of persons with MRSA did not differ substantially from those with methicillin-susceptible *S. aureus* (MSSA). Recent antimicrobial drug use and recent health care were not associated with an increase in MRSA infections. For example, the proportion of persons with recent health care exposure from whom MRSA was isolated (23/262, 9%) did not differ significantly from the proportion of persons without recent health care exposure (11/137, 8%).

Participants lived in 165 villages around Samoa. We detected *S. aureus* in participants from 102 villages. In terms of place of domicile, MRSA was widespread throughout Samoa and was isolated from persons from 27 villages. There did not appear to be any geographic clustering associated with residence close to the main public hospital in Apia, although MRSA was more likely to be isolated from residents of Upolu, the main island on which Apia is located, than from residents of Savai’i, the less-populated and less-developed island. MRSA was isolated from 11% of participants from Upolu, but from only 3% of participants from Savai’i.

Of the 153 MSSA isolates from 187 participants, 124 (81%) isolates were resistant to penicillin, but resistance to any other antimicrobial drug was uncommon. Of the 34 MRSA isolates, 22 were resistant only to β-lactams. Of the remaining 12 MRSA, 8 were ciprofloxacin and erythromycin resistant, 2 were erythromycin resistant with inducible clindamycin resistance, and 2 were ciprofloxacin resistant. All MRSA isolates were susceptible to cotrimoxazole, doxycycline, gentamicin, and vancomycin, and none had constitutive clindamycin resistance.

Except for the MRSA isolates identified as type ST1 by multilocus sequence typing, all other MRSA belonged to a known MRSA strain (Table 2). Seven of the 9 ST1 isolates had indistinguishable PFGE profiles. The ST1 MRSA isolates had 82% homology by PFGE typing with another ST1 MRSA strain common in the region (the Australian WA MRSA-1).

**Table 2:** Strains identified among the MRSA isolates, Samoa, summer 2007–2008*

No. (%) isolates	Strain†	MLST type	No. (%) PVL-positive isolates	*spa* type (% of strain)	Antimicrobial drug susceptibility (% of strain)
10 (29)	USA300	ST8	9 (90)	t008 (100)	Resistant to ciprofloxacin and erythromycin (80); resistant to ciprofloxacin (20)
9 (26)	Queensland clone	ST93	9 (100)	t3949 (56), t202 (44)‡	Resistant only to β-lactams (100)
9§ (26)	–	ST1	0	t1853 (78), t6080 (11)¶	Resistant only to β-lactams (89); resistant to erythromycin** (11)
4 (12)	Southwest Pacific/ WSPP/Oceania clone	ST30	4 (100)	t019 (100)	Resistant only to β-lactams (100)
2 (6)	AK3	ST5	0	t002 (50), t1265 (50)#	Resistant to erythromycin** (50); resistant only to β-lactams (50)

*MRSA, methicillin-resistant *S. aureus*; MLST, multilocus sequence typing; PVL, Panton-Valentine leukocidin; WSPP, Western Samoa Phage Pattern. †International MRSA strain designations, except for AK3, which is a New Zealand designation for a community-associated MRSA strain common in New Zealand. ‡*spa* type t3949 (11–17–23–17–17–**17**–16–16–25) is a single-repeat variant of t202 (11–17–23–17–17–16–16–25). The extra repeat is shown in **boldface**. §Only 8/9 ST1 MRSA isolates were available for PVL PCR, spa typing, and pulsed-field gel electrophoresis (PFGE) typing. Seven of the 8 isolates had indistinguishable PFGE profiles, and the eighth shared 87% homology. ¶*spa* type t6080 (07–23–21–17–13–**13**–34–16–13–33–13) is a single-repeat variant of t1853 (07–23–21–17–13–34–16–13–33–13). The extra repeat is shown in **boldface**. #*spa* type t1265 (26–23–17–34–17–20–17–12–**12**–**12**–16) is a variant of t002 (26–23–17–34–17–20–17–12–**17**–16). The differing repeats are shown in **boldface**. **This isolate also had inducible clindamycin resistance.

Antimicrobial drug resistance profile was associated with strain (Table 2). All isolates of the USA300 strain were ciprofloxacin resistant, and most also were erythromycin resistant. All of the Queensland clone and Southwest Pacific clone MRSA, and 8 of the 9 ST1 MRSA isolates, were resistant only to β-lactams.

## Discussion

Considerable concern exists internationally about increasing levels of antimicrobial drug resistance (*25*). Consequences include treatment failure, the need to use newer antimicrobial drugs to achieve treatment goals, the adverse effects frequently associated with these newer drugs, increased expenditure, and longer hospital stays. In developing countries, such as Samoa, an additional concern exists: when resistance to less expensive antimicrobial drugs becomes widespread, the more expensive antimicrobial drugs are simply not available (*26*). As in many other developing countries (*27*), use of antimicrobial drugs is extremely high in Samoa. More than 60% of all prescriptions dispensed in hospitals and private pharmacies include an antimicrobial drug (*28*). No current data are available on the prevalence of antimicrobial drug resistance in Samoa. In other countries, high levels of antimicrobial drug use correlate with high levels of resistance (*29*). In addition, inadequate dosing and poor adherence by patients may also increase the development of resistance (*25*). Anecdotal evidence indicates these practices also are common in Samoa.

In Samoa, children <5 years of age were more likely than persons in other age groups to have skin and soft tissue infection caused by *S. aureus*; however, the prevalence of MRSA infections were similar in children, adults, and elderly persons. Participants who had reported recent antimicrobial drug use were less likely to have *S. aureus*, but their prevalence of MRSA did not differ significantly from those who did not report recent antimicrobial drug use. The lack of association between MRSA prevalence and health care exposure or antimicrobial drug use was surprising; however, the data on antimicrobial drug use may not be entirely reliable. A previous study found that many Samoans are unclear about which medicines were antimicrobial drugs (*30*). Most of the health care exposure reported in our study related to primary care exposure, and most of the MRSA strains isolated are typically associated with community acquisition (*31*). Even though most participants had community-onset skin and soft tissue infection, more detailed information about diagnosis and place of acquisition would have been valuable.

Few data are available on the prevalence of MRSA in the community in nonindustrialized countries. The World Health Organization has identified a need to strengthen monitoring of antimicrobial drug prescriptions and resistance and has funded pilot programs to achieve this (*25*,*32*). In this study in Samoa, MRSA was isolated from 9% of all participants, and 18% of participants from whom *S. aureus* was isolated had MRSA. Although we attempted to include a wide range of persons in the study, we cannot be sure that some groups in our sample were not overrepresented or underrepresented. Carrying out such research is difficult in a country such as Samoa, where research and laboratory infrastructure are not ideal. Ability to speak Samoan, knowledge of Samoan culture, and previous experience of living in Samoa were essential for the success of the project.

We found that the diversity of MRSA isolates in Samoa that caused skin and soft tissue infection was similar to that in Denmark, a country of >5 million persons (*3*). We expected that isolates of the Southwest Pacific clone MRSA would be the predominant MRSA strain, but these were in the minority. The high prevalence of USA300 and Queensland clone MRSA might reflect the amount of travel between Samoa and the United States and Australia, respectively. The Queensland clone is now common in the Australian states of New South Wales and the Northern Territory in addition to Queensland (*33*). However, the large number of isolates of the Samoa ST1 MRSA clone and the isolation of almost equal numbers of 3 different MRSA clones suggest that the situation may not be that simple.

The Samoa ST1 MRSA isolates were distinct by both PFGE and *spa* typing from the community-associated ST1 MRSA strain commonly found in Australia and New Zealand, WA MRSA-1 (*33*). The Samoa ST1 MRSA isolates shared only 82% homology by PFGE with WA MRSA-1. WA MRSA-1 isolates are typically *spa* type t127, which does not cluster by BURP analysis with the *spa* types (t1853 and t6080) of the Samoa ST1 MRSA. In addition, the WA MRSA-1 strain is characterized by fusidic acid resistance and often also mupirocin or erythromycin resistance, whereas the Samoa ST1 MRSA isolates generally were resistant only to β-lactams. The Samoa ST1 MRSA isolates also were distinct from the USA400 ST1 MRSA strain with which they shared only 80% homology by PFGE. The Samoa ST1 MRSA strain might have originated in the Pacific. However, MRSA with *spa* type t1853 have also been isolated in New Zealand, mainly from patients in the Auckland area, since at least 2008 (*34*).

Three of the 5 strains that we identified among MRSA isolates in Samoa (the USA300, Queensland, and Southwest Pacific strains) typically are associated with community acquisition (*31*). The Samoa ST1 MRSA strain is also likely to be predominantly associated with community-acquired infections. ST1 is recognized as a prominent genetic background of community-associated MRSA (*35*). The Samoa ST1 MRSA shares ≥80% homology by PFGE typing with 2 other ST1 community-associated MRSA strains: WA MRSA-1 and USA400. WA MRSA-1 caused the first cases of community-associated MRSA infection in previously healthy persons in Western Australia in the early 1990s, and USA400 was the strain isolated from the first cases of community-acquired MRSA in the United States (*31*). The Samoa ST1 MRSA strains are not multiresistant, another feature typical of community-associated MRSA strains. On the other hand, none of the isolates of this strain carried the PVL genes, which are commonly found in community-associated MRSA. However, WA MRSA-1 is also PVL negative. Clearly, further characterization of the Samoa ST1 MRSA is warranted.

Evidence suggests that community-associated MRSA strains, particularly USA300, are more easily transmitted and might be more virulent than other *S. aureus* strains (*31*). In North America, approximately one third of persons with community-acquired USA300 MRSA infection require hospital admission (*36*). Moreover, community-associated MRSA is no longer just a problem in the community; it also has become a common cause of health care–associated infections (*37*). Hospital-acquired USA300 infections are more likely than community-acquired USA300 infections to be invasive and be associated with treatment failures (*36*). Thus, increases in the prevalence, severity, and complexity of diseases caused by globally successful community-associated MRSA strains are likely to be associated with increased illness, death, and cost. Economic analyses have consistently demonstrated that MRSA infections are associated with higher cost than are MSSA infections; although these studies have focused primarily on the costs associated with hospital care (*38*). None of these studies have investigated the consequences of MRSA in the developing world, yet the effects of disease are considerable (*39*).

Although reducing MRSA infection in the Samoan community is desirable, no controlled trials have demonstrated effective means of reducing community-associated MRSA infections in a community setting. Recommendations to reduce transmission of community-associated MRSA include washing hands, caring for and covering wounds, not sharing contaminated personal items, appropriately disposing of contaminated waste, and appropriately prescribing antimicrobial drugs (*40*). Any interventions have substantial resource implications for a developing nation but must start with reliable surveillance of antimicrobial drug susceptibility, which is essential to monitor, control, and manage antimicrobial drug resistance. Thus, there is a clear need to assist developing countries with performing quality antimicrobial drug susceptibility testing and surveillance.

The results of our study, together with future surveillance efforts, can be used to provide information for local prescribing; the prevalence of MRSA in Samoa is high, and empiric prescription of antimicrobial drugs needs to account for this high prevalence. For example, we advocate that any patient in Samoa suspected to have serious, invasive *S. aureus* infection have adequate cultures and antimicrobial drug susceptibility testing performed. *S. aureus* infection in such a patient should be treated with vancomycin and a β-lactamase–stable penicillin drug until laboratory results are available. Patients with uncomplicated skin and soft tissue infections requiring antimicrobial drug treatment should receive cotrimoxazole or, if the patient cannot tolerate sulfonamides, clindamycin. Boils or furuncles should be treated by drainage, infection control, and wound care, with antimicrobial drugs reserved for complications.

We identified a wide range of genotypes of MRSA that were causing wound infections in a small Pacific Island nation. Our hope is that this study will provide a starting point for future research into antimicrobial drug resistance in the Pacific and provide impetus for initiatives to improve antimicrobial drug use in Pacific Island nations. Antimicrobial drug resistance is a global concern that does not respect national boundaries; consequently, countries need to assist each other in addressing the problem.

## References

[1] Vandenesch F, Naimi T, Enright M, Lina G, Nimmo G, Heffernan H, Community-acquired methicillin-resistant *Staphylococcus aureus* carrying Panton-Valentine leukocidin genes: worldwide emergence. Emerg Infect Dis. 2003;9:978–84. 12967497 10.3201/eid0908.030089 PMC3020611

[2] Diekema DJ, Pfaller MA, Turnidge J, Verhoef J, Bell J, Fluit AC, Genetic relatedness of multidrug-resistant, methicillin (oxacillin)–resistant *Staphylococcus aureus* bloodstream isolates from SENTRY Antimicrobial Resistance Surveillance Centers worldwide, 1998. Microb Drug Resist. 2000;6:213–21. 10.1089/mdr.2000.6.213 11144421

[3] Larsen AR, Stegger M, Bocher S, Sorum M, Monnet DL, Skov RL. Emergence and characterization of community-associated methicillin-resistant *Staphylococcus aureus* infections in Denmark, 1999 to 2006. J Clin Microbiol. 2009;47:73–8. 10.1128/JCM.01557-08 18971362 PMC2620878

[4] Scribel LV, Silva-Carvalho MC, Souza RR, Superti SV, Kvitko CH, Figueiredo AM, Clinical and molecular epidemiology of methicillin-resistant *Staphylococcus aureus* carrying SCC*mec*IV in a university hospital in Porto Alegre, Brazil. Diagn Microbiol Infect Dis. 2009;65:457–61. 10.1016/j.diagmicrobio.2009.08.012 19766425

[5] Centers for Disease Control and Prevention. Community-associated methicillin-resistant *Staphylococcus aureus* infections in Pacific Islanders—Hawaii, 2001–2003. MMWR Morb Mortal Wkly Rep. 2004;53:767–70. 15329653

[6] Estivariz CF, Park SY, Hageman JC, Dvorin J, Melish MM, Arpon R, Emergence of community-associated methicillin resistant *Staphylococcus aureus* in Hawaii, 2001–2003. J Infect. 2007;54:349–57. 10.1016/j.jinf.2006.08.002 16989904

[7] Munckhof WJ, Schooneveldt J, Coombs GW, Hoare J, Nimmo GR. Emergence of community-acquired methicillin-resistant *Staphylococcus aureus* (MRSA) infection in Queensland, Australia. Int J Infect Dis. 2003;7:259–64. 10.1016/S1201-9712(03)90104-4 14656416

[8] Rings T, Findlay R, Lang S. Ethnicity and methicillin-resistant *S. aureus* in South Auckland. N Z Med J. 1998;111:151. 9612478

[9] Riley D, MacCulloch D, Morris A. Methicillin-resistant *S. aureus* in the suburbs. N Z Med J. 1998;111:59. 9539922

[10] Castrodale LJ, Beller M, Gessner BD. Over-representation of Samoan/Pacific Islanders among patients with methicillin-resistant *Staphylococcus aureus* (MRSA) infections at a large family practice clinic in Anchorage, Alaska, 1996–2000. Alaska Med. 2004;46:88–91. 15999910

[11] Heffernan H, Davies H, Brett M. MRSA increasing in New Zealand. N Z Public Health Rep. 1995;2:97–9.

[12] Łuczak-Kadłubowska A, Sulikowska A, Empel J, Piasecka A, Orczykowska M, Kozinska A, Countrywide molecular survey of methicillin-resistant *Staphylococcus aureus* strains in Poland. J Clin Microbiol. 2008;46:2930–7. 10.1128/JCM.00869-08 18614662 PMC2546761

[13] Robinson DA, Kearns AM, Holmes A, Morrison D, Grundmann H, Edwards G, Re-emergence of early pandemic *Staphylococcus aureus* as a community-acquired methicillin-resistant clone. Lancet. 2005;365:1256–8. 10.1016/S0140-6736(05)74814-5 15811459

[14] Samoa Ministry of Health, Samoa Bureau of Statistics. Samoa Demographic and Health Survey 2009. Apia (Samoa): The Ministry; 2009.

[15] Hill PC, Birch M, Chambers S, Drinkovic D, Ellis-Pegler RB, Everts R, Prospective study of 424 cases of *Staphylococcus aureus* bacteraemia: determination of factors affecting incidence and mortality. Intern Med J. 2001;31:97–103. 10.1111/j.1444-0903.2001.00029.x 11480485

[16] Finger F, Rossaak M, Umstaetter R, Reulbach U, Pitto R. Skin infections of the limbs of Polynesian children. N Z Med J. 2004;117:U847. 15107869

[17] Rossaak M, Pitto R. Osteomyelitis in Polynesian children. Int Orthop. 2005;29:55–8. 10.1007/s00264-004-0597-3 15490163 PMC3456946

[18] Institute of Environmental Science and Research Limited. Antimicrobial resistance data from hospital and community laboratories, 2008 [cited 2010 Nov 20]. http://www.surv.esr.cri.nz/PDF_surveillance/Antimicrobial/AR/National_AR_2008.pdf

[19] Clinical and Laboratory Standards Institute. Performance standards for antimicrobial disk susceptibility tests: approved standard. 9th ed. Wayne (PA): The Institute; 2006.

[20] Clinical and Laboratory Standards Institute. Methods for dilution antimicrobial susceptibility tests for bacteria that grow aerobically: approved standard. 7th ed. Wayne (PA): The Institute; 2006.

[21] Clinical and Laboratory Standards Institute. Performance standards for antimicrobial susceptibility testing; 18th informational supplement. Wayne (PA): The Institute; 2008.

[22] Lina G, Piémont Y, Godail-Gamot F, Bes M, Peter M, Gauduchon V, Involvement of Panton-Valentine leukocidin-producing *Staphylococcus aureus* in primary skin infections and pneumonia. Clin Infect Dis. 1999;29:1128–32. 10.1086/313461 10524952

[23] Enright MC, Day NP, Davies CE, Peacock SJ, Spratt BG. Multilocus sequence typing for characterization of methicillin-resistant and methicillin-susceptible clones of *Staphylococcus aureus.* J Clin Microbiol. 2000;38:1008–15. 10698988 10.1128/jcm.38.3.1008-1015.2000 PMC86325

[24] Strommenger B, Braulke C, Heuck D, Schmidt C, Pasemann B, Nubel U, *spa* typing of *Staphylococcus aureus* as a frontline tool in epidemiological typing. J Clin Microbiol. 2008;46:574–81. 10.1128/JCM.01599-07 18032612 PMC2238071

[25] World Health Organization. WHO global strategy for containment of antimicrobial resistance. Geneva: The Organization; 2001.

[26] Okeke IN, Laxminarayan R, Bhutta ZA, Duse AG, Jenkins P, O’Brien TF, Antimicrobial resistance in developing countries. Part I: recent trends and current status. Lancet Infect Dis. 2005;5:481–93. 10.1016/S1473-3099(05)70189-4 16048717

[27] Hart CA, Kariuki S. Antimicrobial resistance in developing countries. BMJ. 1998;317:647–50. 9727995 10.1136/bmj.317.7159.647 PMC1113834

[28] Norris P, Nguyen H. Consumption of antibiotics in a small Pacific Island nation: Samoa. Pharmacy Practice. 2007;5:36–41. 25214916 10.4321/s1886-36552007000100006 PMC4155148

[29] Goossens H, Ferech M, Vander Stichele R, Elseviers M. Outpatient antibiotic use in Europe and association with resistance: a cross-national study. Lancet. 2005;365:579–87. 15708101 10.1016/S0140-6736(05)17907-0

[30] Norris P, Vaai C, Faalau F, Churchward M, Arroll B. Pain, infection and colds and flu: Samoan people’s views about antibiotics. Res Social Adm Pharm. 2011;7:81–92. 10.1016/j.sapharm.2010.01.002 21397883

[31] Deleo FR, Otto M, Kreiswirth BN, Chambers HF. Community-associated meticillin-resistant *Staphylococcus aureus.* Lancet. 2010;375:1557–68. 10.1016/S0140-6736(09)61999-1 20206987 PMC3511788

[32] Holloway K. Community-based surveillance of antimicrobial use and resistance in resource-constrained settings. Geneva: World Health Organization; 2009.

[33] Nimmo GR, Coombs GW. Community-associated methicillin-resistant *Staphylococcus aureus* (MRSA) in Australia. Int J Antimicrob Agents. 2008;31:401–10. 10.1016/j.ijantimicag.2007.08.011 18342492

[34] Richardson A, Pope C, Couper J, Desai U, Heffernan H. Annual survey of methicillin-resistant *Staphylococcus aureus* (MRSA), 2009 [cited 2010 Nov 20]. http://www.surv.esr.cri.nz/PDF_surveillance/Antimicrobial/MRSA/aMRSA_2009.pdf.

[35] David MZ, Daum RS. Community-associated methicillin-resistant *Staphylococcus aureus*: epidemiology and clinical consequences of an emerging epidemic. Clin Microbiol Rev. 2010;23:616–67. 10.1128/CMR.00081-09 20610826 PMC2901661

[36] Moore CL, Hingwe A, Donabedian SM, Perri MB, Davis SL, Haque NZ, Comparative evaluation of epidemiology and outcomes of methicillin-resistant *Staphylococcus aureus* (MRSA) USA300 infections causing community- and healthcare-associated infections. Int J Antimicrob Agents. 2009;34:148–55. 10.1016/j.ijantimicag.2009.03.004 19394801

[37] Tenover FC. Community-associated methicillin-resistant *Staphylococcus aureus*: it’s not just in communities anymore. Clin Microbiol Newsl. 2006;28:33–6. 10.1016/j.clinmicnews.2006.02.001

[38] Gould IM, Reilly J, Bunyan D, Walker A. Costs of healthcare associated methicillin-resistant *Staphylococcus aureus* (MRSA) and its control. Clin Microbiol Infect. 2010;16:1721–8. 10.1111/j.1469-0691.2010.03365.x 20825434

[39] Nickerson EK, Hongsuwan M, Limmathurotsakul D, Wuthiekanun V, Shah KR, Srisomang P, *Staphylococcus aureus* bacteraemia in a tropical setting: patient outcome and impact of antibiotic resistance. PLoS ONE. 2009;4:e4308. 10.1371/journal.pone.0004308 19180198 PMC2628727

[40] Barton M, Hawkes M, Moore D, Conly J, Nicolle L, Upton A, Guidelines for the prevention and management of community-associated methicillin-resistant *Staphylococcus aureus*: a perspective for Canadian health care practitioners. Can J Infect Dis Med Microbiol. 2006;17(Suppl C):4–24C. PMC3555463 23365589

